# Valuable effects of *lactobacillus* and citicoline on steatohepatitis: role of Nrf2/HO-1 and gut microbiota

**DOI:** 10.1186/s13568-023-01561-8

**Published:** 2023-06-08

**Authors:** Ahmed M. El-Baz, Amira M. El-Ganiny, Doaa Hellal, Hala M. Anwer, Hend A. Abd El-Aziz, Ibrahim E. Tharwat, Mohamed A. El-Adawy, Shehab El-Din M. Helal, Menna Tallah A. Mohamed, Tassnim M. Azb, Hanya M. Elshafaey, AbdulRahman A. Shalata, Sahar M. Elmeligi, Noran H. Abdelbary, Attalla F. El-kott, Fatimah A. Al-Saeed, Eman T. Salem, Mohamed M. Adel El-Sokkary, Ahmed Shata, Ahmed A. Shabaan

**Affiliations:** 1 Department of Microbiology and Biotechnology, Faculty of Pharmacy, Delta University for Science and Technology, 11152 Gamasa, Egypt; 2 Department of Microbiology and Biotechnology, Faculty of Pharmacy, Delta University for Science and Technology, International Coastal Road, Gamasa City, Mansoura, Dakahlia P.O. Box +11152, Egypt; 3 Department of Microbiology and Immunology, Faculty of Pharmacy, Zagazig University, 44519 Zagazig, Egypt; 4 Department of Clinical Pharmacology, Faculty of Medicine, Mansoura University, 35516 Mansoura, Egypt; 5 Department of Physiology, Faculty of Medicine, Benha University, Benha, Egypt; 6 Department of Clinical Pharmacy, Faculty of Pharmacy, Delta University for Science and Technology, 11152 Gamasa, Egypt; 7 Department of Biology, College of Science, King Khalid University, 61421 Abha, Saudi Arabia; 8 Department of Zoology, College of Science, Damanhour University, 22511 Damanhour, Egypt; 9 Department of Basic Science, Faculty of Physical Therapy, Horus University-Egypt, 34518 Horus, New Damietta Egypt; 10 Microbiology Department, Faculty of Pharmacy, Mansoura University, 35516 Mansoura, Egypt; 11 Department of Pharmacology, Faculty of Pharmacy, Delta University for Science and Technology, 11152 Gamasa, Egypt; 12 Department of Pharmacology and Toxicology, Faculty of pharmacy, Mansoura University, 35516 Mansoura, Egypt

**Keywords:** NASH, *Lactobacillus*, Citicoline, Nrf2/HO-1, TLR4/NF-kB, Gut microbiota

## Abstract

Non-alcoholic steatohepatitis (NASH) is a more dangerous form of chronic non-alcoholic fatty liver disease (NAFLD). In the current investigation, the influence of citicoline on high-fat diet (HFD)-induced NASH was examined, both alone and in combination with *Lactobacillus* (probiotic). NASH was induced by feeding HFD (10% sugar, 10% lard stearin, 2% cholesterol, and 0.5% cholic acid) to rats for 13 weeks and received single i.p. injection of streptozotocin (STZ, 30 mg/kg) after 4 weeks. Citicoline was given at two dose levels (250 mg and 500 mg, i.p.) at the beginning of the sixth week, and in combination with an oral suspension of *Lactobacillus* every day for eight weeks until the study’s conclusion. HFD/STZ induced steatohepatitis as shown by histopathological changes, elevated serum liver enzymes, serum hyperlipidemia and hepatic fat accumulation. Moreover, HFD convinced oxidative stress by increased lipid peroxidation marker (MDA) and decreased antioxidant enzymes (GSH and TAC). Upregulation of TLR4/NF-kB and the downstream inflammatory cascade (TNF-α, and IL-6) as well as Pentaraxin, fetuin-B and apoptotic markers (caspase-3 and Bax) were observed. NASH rats also had massive increase in *Bacteroides spp., Fusobacterium spp., E. coli, Clostridium spp., Providencia spp., Prevotella interrmedia*, and *P. gingivalis* while remarkable drop in *Bifidobacteria spp*. and *Lactobacillus spp.* Co-treatment with citicoline alone and with *Lactobacillus* improve histopathological NASH outcomes and reversed all of these molecular pathological alterations linked to NASH via upregulating the expression of Nrf2/HO-1 and downregulating TLR4/NF-kB signaling pathways. These results suggest that citicoline and lactobacillus may represent new hepatoprotective strategies against NASH progression.

**Supplementary Information:**

The online version contains supplementary material available at 10.1186/s13568-023-01561-8.

## Introduction

Non-alcoholic fatty liver disease (NAFLD) is defined as accumulation of fats in hepatocytes that exceeds 5% of liver weight in a patient who is not alcoholic (Vernon et al. 2011). NAFLD is the most common cause of chronic liver disease in developed countries (Younossi et al. 2016). It can affect children, adolescents, and the elderly (Jiang et al. 2020). NAFLD incidence is assessed to be around 25% worldwide, with higher rate (31.79%) was reported in the Middle East (Younossi et al. 2016). It covers a wide range of histopathological conditions, from simple steatosis to non-alcoholic steatohepatitis (NASH) which can lead to cirrhosis and hepatocellular carcinoma (HCC). HCC is one of the foremost causes of cancer-related deaths worldwide (Dhamija et al. 2019).

Obesity, metabolic syndromes, and diabetes mellitus are common risk factors of emerging NAFLD (Yilmaz and Eren 2017). While only a small percentage of patients with simple steatosis (earliest form of NAFLD) progress to NASH (2–5%). A number of those patients develop NASH-related complications (cardiovascular disease, advanced fibrosis, cirrhosis, and HCC) which place a significant load on the healthcare system (Yilmaz and Eren 2017).

The development of steatosis to NASH can be attributed to the effect of oxidative stress which cause hepatocytes injury. Furthermore, changes in the gut microbiota have been related with NASH pathogenesis and progression (Carter et al. 2021). The release of endotoxins (LPS) of Gram negative bacteria stimulate the immune system-to produce a pro-inflammatory cytokines principal to mitochondrial dysfunction (Braunersreuther et al. 2012).

NASH can be diagnosed by liver biopsy and histopathology (Vernon et al. 2011). Several non-invasive biomarkers can also be measured, for example, oxidative stress and elevated mitochondrial permeability lead to caspase activation. Oxidative stress causes lipid peroxidation, producing malondialdehyde (MDA). The effects of ROS were mitigated by antioxidants, which can be classified as enzymatic or non-enzymatic. Enzymatic antioxidants include superoxide dismutase (SOD), which converts superoxide anion to hydrogen peroxide, whereas catalase (CAT) neutralizes hydrogen peroxide in water. In contrast, non-enzymatic forms comprise a wide range of molecules, such as glutathione. These products serve as helpful in vivo indicators for oxidative stress (Phaniendra et al. 2015). In addition, the diagnosis of NASH is aided by raised liver enzymes such as alanine aminotransferase (ALT) and aspartate aminotransferase (AST), as well as higher cytokines like tumor necrosis factor (TNF)-alpha and interleukin (IL)-6. (Neuman et al. 2014).

Biologically, the liver and gut are anatomically linked via a bidirectional functional link called the gut-liver axis (GLA). Because of this strong relationship, changes in gut microbiota might alter the liver’s state (Mazzotti et al. 2016). Dysbiosis of gut microbiota is defined as an “imbalance” in the gut microbial community due to gain or loss of a member or a change in relative abundance of microbes (Hrncir 2022). Gut microbiota dysbiosis is linked to numerous intestinal and extra-intestinal diseases including NAFLD (Rinninella et al. 2019). According to a recent study, NAFLD have less variety in gut microbiota than healthy people (Saltzman et al. 2018).

During the past few years, new technologies have facilitated identification and quantification of gut microbiota components by analyzing nucleic acids (DNA and RNA). In the gut of healthy people, the most prevalent bacterial phyla are Bacteroidetes (65.2%), Firmicutes (29.6%), Proteobacteria (2.9%), and Actinobacteria (0.5%) (Jennison and Byrne 2021). *Prevotella* and *Bacteroides* are two of the most common genera in the family Bacteroidetes. Numerous genera, including *Lactobacillus*, *Bacillus*, *Clostridium*, and *Enterococcus*, are members of the Firmicutes phylum. The *Bifidobacterium* genus is primarily responsible for representing the Actinobacteria phylum (Arumugam et al. 2011). Because the gut microbiota is thought to have a role in NAFLD, researchers have recently been excited in finding drugs that can regulate NAFLD by modifying the gut dysbiosis (Jiang et al. 2020).

Probiotics are living microorganisms that help the host’s health by restoring normal microbiota and relieving digestive disorders (Jiang et al. 2020). Probiotics have the potential to boost human immunity, especially when used in the right doses. Lactic acid bacilli have been shown to have immune-modulating effects on chronic inflammation due to changes in the gut microbiome (El-Baz et al. 2021). Many *Lactobacillus species* have been shown to alter gut microbiota dysbiosis by modifying the gut microbiota environment (El-Baz et al. 2020). NAFLD patients treated with *Lactobacillus bulgaricus* and *Streptococcus thermophiles* had lower levels of liver aminotransferases, according to a human clinical trial (Aller et al. 2011). Furthermore, another study suggested that *Lactobacillus* and *Bifidobacterium* probiotics can be effective in improving pediatric NAFLD (Famouri et al. 2017).

The consequence of choline deficiency on the progress of NAFLD is well known (Corbin and Zeisel 2012). Citicoline is a food supplement that is considered as a source of choline. Choline helps support healthy lipid metabolism and keep the liver functioning normally (Synoradzki and Grieb 2019). It can improve hepatic encephalopathy through reduction of oxidative stress (Iulia et al. 2017). As the prevalence of NAFLD has increased to more than 30% of adults in developed countries and its incidence is still on rise.

The goal of the current study was to evaluate Nrf2/HO-1 and TLR4/NF-kB signaling pathways, as well as identify the dysbiosis in the gut microbiome that was associated with experimentally induced NASH, in order to investigate the potential therapeutic effects of citicoline either alone or in combination with *Lactobacillus* in experimentally induced NASH.

## Materials and methods

### Chemicals and reagents

Citicoline was supplied as sterile ampoules containing 500 mg/4 mL (Somazina, Ferrer International S.A., Spain). Lactobacillus was obtained as packets of Lacteol forte (Rameda Pharma Co, Giza, Egypt). STZ, carboxymethyl cellulose, cholesterol and cholic acid were acquired from Sigma Chemical Co. (St. Louis, Missouri, USA).

### Animals

Male adult Sprague Dawley rats (180–220 g, n = 6–8) supplied by delta university, Mansoura (Egypt) were utilized. Prior to the start of the experiment, rats were acclimated for a week. Throughout the trial, rats were kept under conventional dietary and environmental circumstances. Research Ethics Committee at Faculty of Pharmacy, Delta University approved the experimental protocol (ethical approval number: FPDU6/2022).

### Induction of fatty liver in rats

**Fig. 1:**
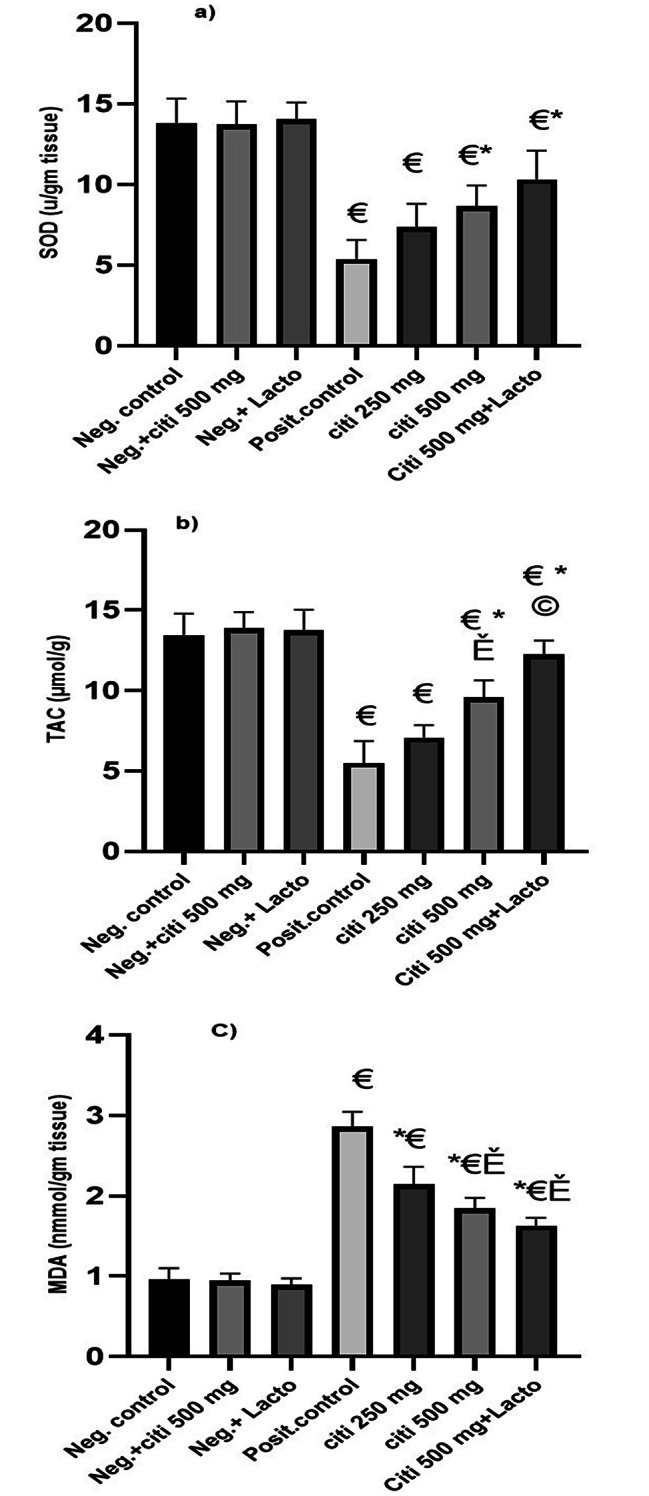
Effect of the tested monotherapies and therapeutic combinations on oxidative stress markers in fatty liver tissues: (**a**) SOD (U/g tissue), (**b**) TAC (µmol/g tissue), (**c**) MDA (nmmol/g tissue). Data are expressed as means ± SEM. Results are considered significant when P < 0.05. Neg. control: Control group; Neg.+ citi 500: Control + citicoline 500 mg group; Neg.+ Lacto: Control + Lactobacillus; Posit. control: HFD (Fatty liver) group, citi 250 mg: HFD + citicoline 250 mg group; citi 500 mg: HFD + citicoline 500 mg group; citi + Lacto: HFD + citicoline 500 mg + *Lactobacillus* group. *vs positive group, € vs. negative group, Ě vs. citicoline 250 mg, © vs. citicoline 500

Rats were given a high-fat diet (HFD) for 13 weeks that contained 10% sugar, 10% lard stearin, 2% cholesterol, and 0.5% cholic acid. After four weeks, rats received an IP injection of STZ (30 mg/kg in citrate buffer, 1 mL/kg). Seven days following STZ injection, fasting blood glucose (FBG) levels were measured. Only rats with FBG levels above 200 mg/dL were classified as diabetic and permitted to finish the experiment. After STZ treatment, the HFD remained unchanged, but sugar was removed to prevent severe hyperglycemia (El-Derany and El-Demerdash, 2020). The study’s eight-week treatment period started at the start of the sixth week and continued until it was through (Fig. 1S).

### Experimental design

The animals were divided into seven groups that include: (1) Control group (Neg. Control): rats were fed normal chow diet, (2) Citicoline group (Neg. + citi 500 mg): rats were fed normal chow diet and administered citicoline (500 mg/kg, IP). (3) Lactobacillus group (Neg.+ Lacto): rats were fed normal chow diet and administered lactobacillus, the Lacteol forte packet’s contents were dissolved in water to create the probiotic suspension, which contained *Lactobacillus fermentum and Lactobacillus delbrueckii.* (Garcia-Castillo et al. 2019). In order to offer the probiotic-treated groups 0.5 mL of microbial suspension containing 2.7 × 10^8^ CFU/mL, the concentration of the solution was diluted from the starting concentration of 1 × 10^10^ microbial cells per package. This was ingested by rats using a gastric tube (Fooladi et al. 2015). (4) HFD group (Posit. Control): rats were fed HFD as mentioned above. (5) Citicoline 250 mg (Citi 250) + HFD: rats were fed HFD and received citicoline (250 mg/kg) beginning with the start of the sixth week and lasting for 8 weeks until the study’s conclusion. (6) Citicoline 500 mg (Citi 500) + HFD: rats were fed HFD and received citicoline (500 mg/kg) beginning with the start of the sixth week and lasting for 8 weeks until the study’s conclusion. (7) Citicoline + Lactobacillus (Citi 500 + Lacto) group + HFD: rats were fed HFD and received citicoline (500 mg/kg) and lactobacillus beginning with the start of the sixth week and lasting for 8 weeks until the study’s conclusion.

### Animal sacrifice and sample collection

Rats were deprived overnight after the experiment, and the next day they were weighed and given thiopental to make them unconscious. Serum samples were separated and stored at -80 °C for analysis after blood samples were taken through cardiac puncture and centrifuged at 3000 rpm for 20 min. All animals’ livers were also removed, cleaned, weighed, and cleansed with ice-cold phosphate buffered saline (PBS, pH 7.4). Parts of liver tissues were homogenized (10% w/v) in 20 mM Tris–HCl (containing 1 mM EDTA, pH 7.4) and centrifuged at 3000 g for 20 min at 4 °C. The supernatants were collected and stored at -80 °C for subsequent biochemical analyses. Portions of livers were fixed in 10% neutral buffered formalin solutions to be used for histopathological examination. Additionally, stool samples were obtained by weighing 300 mg cecal feces samples shortly after the animals were euthanized. The QIAamp DNA Stool Mini Kit (Qiagen Inc., cat. # 51,504 Hilden, Germany) was used to extract DNA according to the manufacturer’s instructions. To measure the DNA concentration, the obtained DNA was examined spectrophotometrically using a NanoDrop device (OPTIZEN NanoQ, Mecasys) and stored at -20 °C. The rest of the stool samples were stored at -80 °C until they were required again.

### Liver injury blood biochemical parameters analysis and liver index

Using commercially available kits from Spectrum Diagnostics (Cairo, Egypt), serum aspartate aminotransferase (AST), alanine aminotransferase (ALT), and alkaline phosphatase (ALP) levels were measured spectrophotometrically. Additionally, the following equation was used to determine liver index: (Hepatic Mass/Total Body Mass) x 100.

### Measurement of serum lipid profile

Using an enzymatic colorimetric method (Biodiagnostics, Cairo, Egypt), serum levels of total cholesterol (TC), triglycerides (TGs), and high-density lipoprotein cholesterol (HDL-C) were measured in accordance with the procedures outlined by Allain et al. (1974), Fossati and Prencipe (1982) and Lopes-Virella et al. (1977), respectively.

### Pentraxin and fetuin-B assays

Fetuin-B and pentraxin 3 (PTX3) plasma concentrations were measured using the rat ELISA Kit (USCN Life Science Inc., USA).

### Hepatic oxidative stress parameters

In the liver homogenates’ supernatants, these parameters were estimated. A measure of lipid peroxidation is malondialdehyde (MDA) level, while measures of the liver’s antioxidant capacity are total antioxidant capacity (TAC) and superoxide dismutase (SOD) activity. The previously stated technique was used to determine MDA content (Ohkawa et al. 1979). In a nutshell, MDA was determined by thiobarbituric acid (TBA) reaction and spectrophotometric measurement of absorbance at 532 nm. Monitoring the previously mentioned SOD-inhabitable auto-oxidation of pyrogallol was used to measure the SOD activity (Marklund 1985). At 420 nm, the change in absorbance was seen. By reacting a known amount of exogenously supplied hydrogen peroxide (H_2_O_2_) with TAC, the amount of TAC was determined. A portion of the supplied hydrogen peroxide is removed by the antioxidants in the sample. Through an enzymatic reaction that turns 3,5, dichloro-2-hydroxy benzensulphonate into a colored product, the residual H_2_O_2_ is measured colorimetrically. At 505 nm, the absorbance was determined using spectrophotometry (Koracevic et al. 2001).

### Cytokines estimation

Levels of TNF-α and IL-6 in the supernatants of liver tissues were determined using ELISA kits according to manufacturer’s protocol (RayBiotech Inc., Norcross, GA, USA; and eBioscience Inc., San Diego, CA, USA) respectively.

### Estimation of hepatic Nrf2 and HO-1 levels

Nuclear factor erythroid 2-related factor (Nrf2) and Heme oxygenase-1 (HO-1) activity were assessed by ELISA assay kits (Cloud-Clone Co., Houston, USA) according to manufacturer’s instructions.

### Estimation of apoptosis markers

The pro-apoptotic markers, Caspase-3 and Bax proteins expression, were evaluated in liver tissue homogenates according to the manufacturers ’instruction, using ELISA kits purchased from (BioVisionInc, Catalog #E4592 Milpitas, USA; and Cusabio, Cat. # CSB-EL002573RA, Houston, USA), respectively.

### Hepatic histopathological and immunohistochemical assessments

Dissected liver tissue samples were fixed in neutral buffered formalin at a 10% concentration. The tissue was processed, and paraffin blocks were created using conventional histopathological methods. Using the Ishak-modified HAI system, fibrosis and necroinflammation alterations were assessed in Sect. (5 μm thick) stained with Mallory’s trichrome and hematoxylin-eosin, respectively (Ishak et al. 1995). Using the following NAFLD activity scores (NAS), the degree of hepatic steatosis, inflammation, and ballooning was evaluated. The specimens were categorized into grades 0–3 for hepatocellular steatosis (grade 0, where less than 5% of the hepatic parenchyma were occupied by steatosis; grade 1, where 6–33% of the hepatic parenchyma were occupied by steatosis; grade 2, where 34–66% of the hepatic parenchyma were occupied by steatosis; and grade 3, where more than 66% of the hepatic parenchyma were occupied by steatosis). The specimens were divided into classes 0–3 according to the degree of inflammatory cell infiltration (grade 0, no infiltration; grade 1, one to two foci per 400X field; grade 2, three to four foci per 400X field; and grade 3, more than four foci per 400X field). The specimens were categorized into grades 0–2 for hepatocellular ballooning (grade 0, no ballooning; grade 1, few balloon cells; and grade 2, many cells/prominent ballooning). Stages 0–4 of hepatic fibrosis were determined (stage 0, no fibrosis; stages 1 and 2, mild and moderate perisinusoidal and periportal fibrosis respectively; stage 3, bridging fibrosis; and stage 4, cirrhosis). Anti-NF-kB and anti-TLR_4_ (Santa Cruz, CA, USA) were utilized as primary antibodies for immunohistochemical investigation in order to identify their intended proteins using conventional immunohistochemistry techniques.

### Real-time PCR test for measuring relative abundance of selected gut microbiota

The relative abundance of *Bifidobacteria, Lactobacillus, Bacteroides Escherichia coli (E. coli)*, *Clostridium*, *Fusobacteria, P. gingivalis, Providencia* and *Prevotella intermedia* were determined using the quantitative real-time polymerase chain reaction (qRT-PCR). Total bacterial abundance was estimated using suitable primers for 16 S rRNA housekeeping gene amplification (Table 1**).** For quantitative DNA measurements, 40–80 ng of extracted faecal DNA was mixed with 12.5 µL (2x) SYBR Green PCR master mix (Willowfort Co., Birmingham, UK.) and 1.5 µL of each forward and reverse primer (10 µmol each). To make a final volume of 25 µL, 7.5 µL of nuclease-free H_2_O was added. On a MyGo real-time PCR system, all real-time reactions were done at 95 °C for 5 min, followed by 45 cycles of 95 °C for 20 s, annealing for 20 s (Table 1), then 72 °C for 40 s and final extension at 72 °C for 5 min (Wong et al. 2017).

**Table 1:** Different primers used in this study to detect different species of gut bacteria

Primer name	Primer Sequence		Annealing Temp.	Size bp	Reference
*All bacteria*	F	GAGTTTGATCCTGGCTCAG	51	312	(Ginige et al. 2013)
	R	GCTGCCTCCCGTAGGAGT			
*Porphyromonas gingivalis*	F	AATCGTAACGGGCGACACAC	53	594	(Benkirane et al. 1995)
	R	GGGTTGCTCCTTCATCACAC			
*Fusobacterium spp.*	F	GGATTTATTGGGCGTAAAGC	51.5	162	(Castellarin et al. 2012)
	R	GGCATTCCTACAAATATCTACGAA			
*E. coli*	F	TGGGAGCGAAAATCCTG	47.5	219	(Maheux et al. 2009)
	R	CAGTACAGGTAGACTTCTG			
*Providencia spp.*	F	ACCGCATAATCTCTTAGG	43.5	514	(Burns et al. 2015)
	R	CTACACATGGAATTCTAC			
*Bifidobacterium spp.*	F	CTCCTGGAAACGGGTGG	51	551	(Matsuki et al. 2002)
	R	GGTGTTCTTCCCGATATCTACA			
*Prevotella intermedia*	F	CGAACCGTCAAGCATAGGC	54	368	(Kim et al. 2011)
	R	AACAGCCGCTTTTAGAACACAA			
*Bacteroides spp.*	F	AAGGGAGCGTAGATGGATGTTTA	55	193	(Huijsdens et al. 2002)
	R	CGAGCCTCAATGTCAGTTGC			
*Clostridium spp.*	F	CGGTACCTGACTAAGAAGC	50	429	(Bartosch et al. 2004)
	R	AGTTTGATTCTTGCGAACG			
***Lactobacillus spp.***	F	AGCAGTAGGGAATCTTCCA	50	334	(Collado et al. 2008)
	R	CACCGCTACACATGGAG			

### Statistical analysis

One-way analysis of variance (ANOVA) and the Tukey’s Kramer Multiple Comparison Test were used to assess the data. The non-parametric Kruskal-Wallis test and Dunn’s multiple comparison post-test were used to analyse the histopathological scores. The data are represented as means and standard error of the mean (SEM). Significant was defined as p < 0.05.

## Results

### Effect of citicoline and its combination with *Lactobacillus* on HFD induced oxidative stress

MDA levels rose following HFD in comparison to control rats, a marker of oxidative stress. Additionally, reduced levels of SOD and TAC indicated that the liver’s capacity to function as an antioxidant was compromised. However, either alone or in combination with *Lactobacillus*, citicoline significantly increased SOD and TAC levels in comparison to the NASH group. Levels of MDA were also reduced. Additionally, the effect of Citi 500 on MDA is noticeably larger as compared to Citi 250. (p ˂0.05). Except for its impact on TAC, there is no noticeable difference between *Lactobacillus* and the aforementioned criteria. (p˂0.01) (Fig. 1).

### Effect of citicoline and its combination with *Lactobacillus* on biochemical markers of liver injury and liver index

**Fig. 2:**
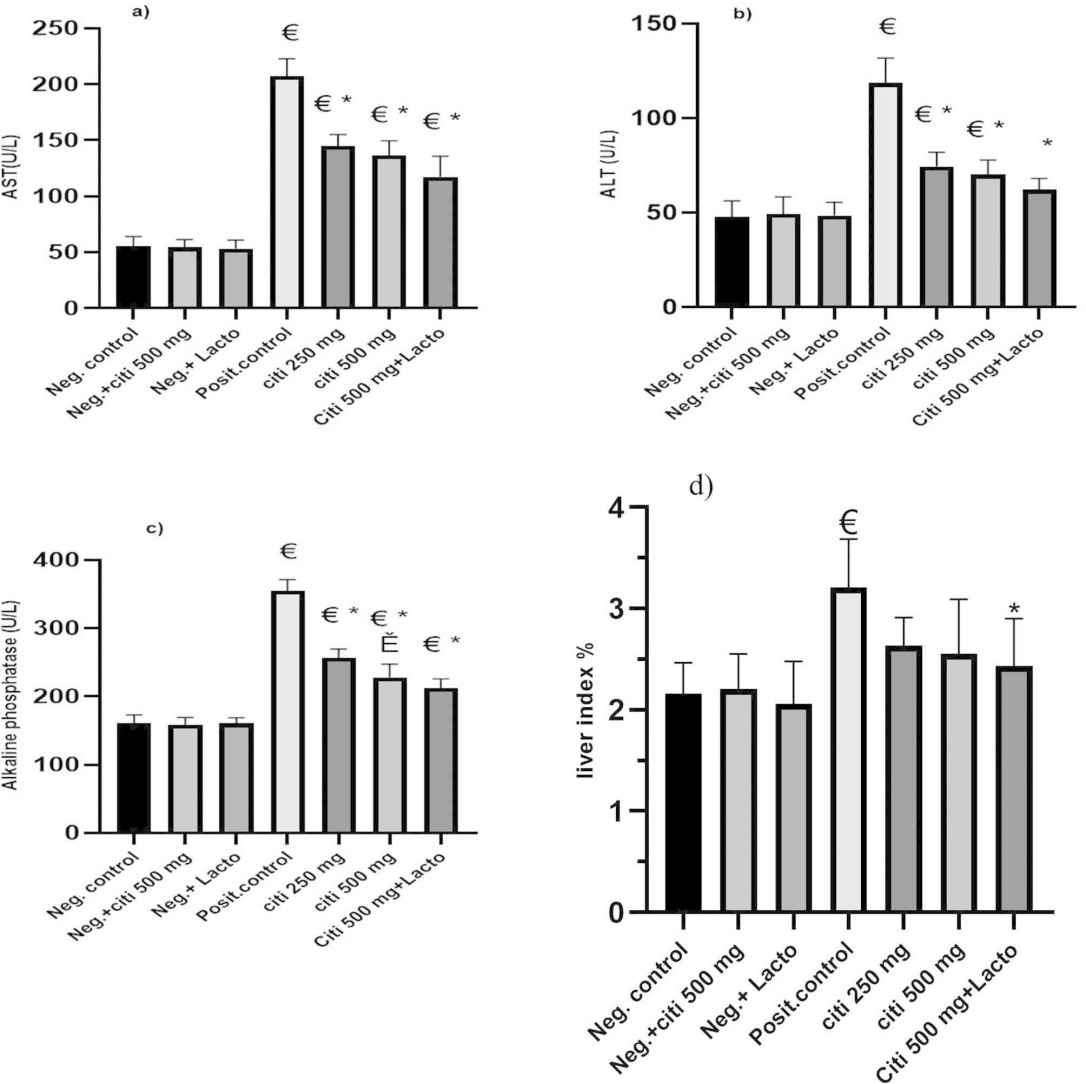
Effect of the tested monotherapies and therapeutic combinations on serum level of liver enzymes and liver index: (**a**) AST (U/L), (**b**) ALT (U/L), (**c**) ALP (U/L), (**d**) liver index. Data are expressed as means ± SEM. Results are considered significant when P < 0.05. Neg. control: Control group; Neg.+ citi 500: Control + citicoline 500 mg group; Neg.+ Lacto: Control + Lactobacillus; Posit. control: HFD (Fatty liver) group, citi 250 mg: HFD + citicoline 250 mg group; citi 500 mg: HFD + citicoline 500 mg group; citi + Lacto: HFD + citicoline 500 mg + *Lactobacillus* group. *vs positive group, € vs. negative group, Ě vs. citicoline 250 mg, © vs. citicoline 500

When compared to the control group, the NASH group had severe liver damage after 13 weeks as evidenced by a marked increase in blood ALT, AST, and ALP levels. (Fig. 2). Comparing the citicoline-treated groups to the NASH group, it was interesting to see that the levels of ALT, AST, and ALP decreased in a dose-dependent manner. In comparison to the citi 500 group, the modification brought forth by the addition of *Lactobacillus* is not noteworthy. Contrarily, liver index greatly decreased after treatment with the combination of citicoline and *Lactobacillus* compared to the HFD group, while liver index dramatically rose in the HFD group compared to the control group (Fig. 2).

### Effect of citicoline and its combination with *Lactobacillus* on IL-6 and TNF-α

**Fig. 3:**
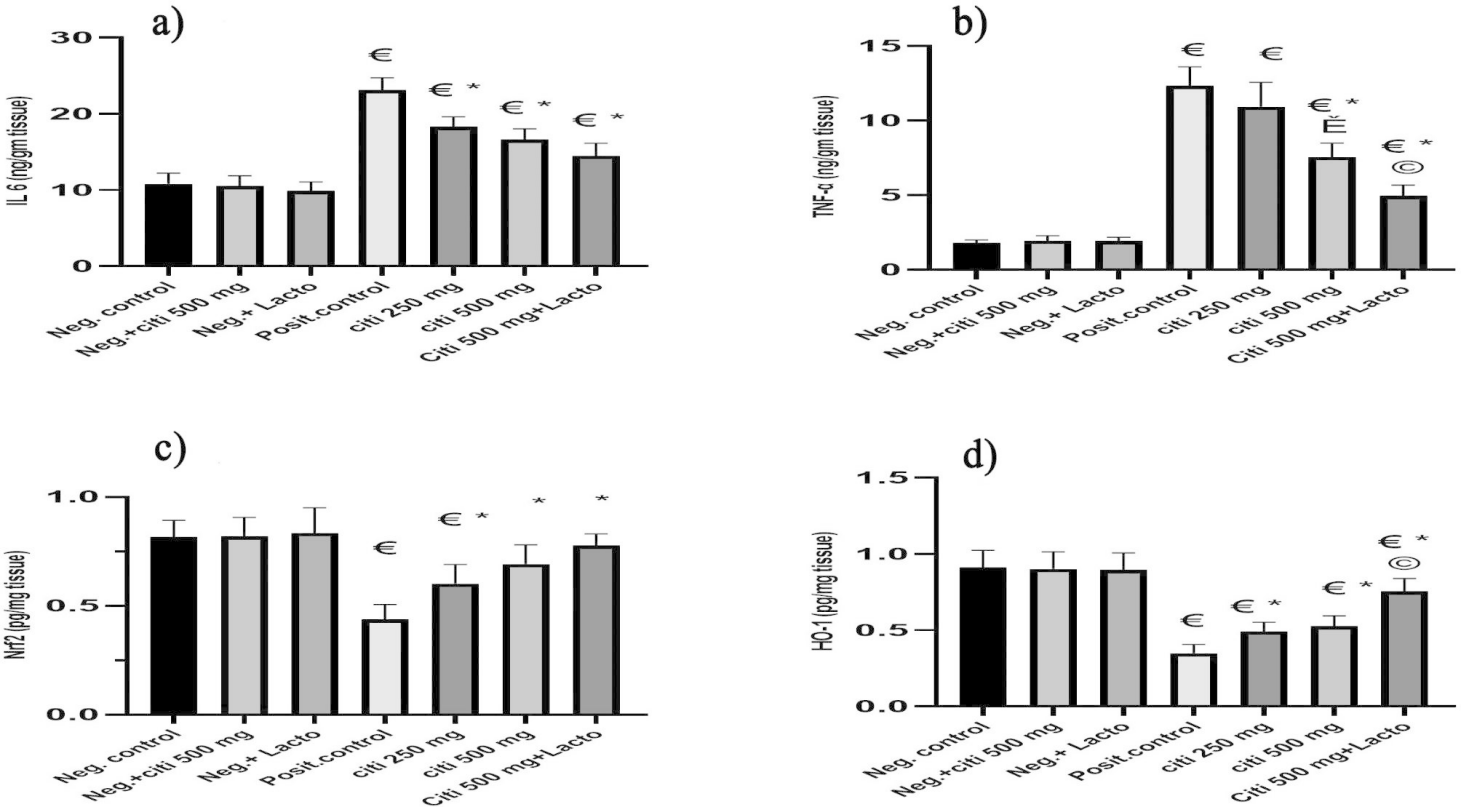
Effect of the tested monotherapies and therapeutic combinations on pro-inflammatory in fatty liver tissues: (**a**) IL6 (ng/g tissue), (**b**) TNF-α (ng/g tissue), (**c**) Nrf2 (pg/mg tissue), (**d**) HO-1 (pg/mg tissue). Data are expressed as means ± SEM. Results are considered significant when P < 0.05. Neg. control: Control group; Neg.+ citi 500: Control + citicoline 500 mg group; Neg.+ Lacto: Control + Lactobacillus; Posit. control: HFD (Fatty liver) group, citi 250 mg: HFD + citicoline 250 mg group; citi 500 mg: HFD + citicoline 500 mg group; citi + Lacto: HFD + citicoline 500 mg + *Lactobacillus* group. *vs positive group, € vs. negative group, Ě vs. citicoline 250 mg, © vs. citicoline 500

In comparison to the rats of a negative control, HFD increased the levels of IL-6 and TNF-α in the liver tissue. As seen in Fig. 3a, all drugs under consideration significantly inhibit IL-6 (p˂0.0001, p˂0.0001, and p˂0.0001). Citi 500 and its combination with *Lactobacillus* substantially reduce TNF- (p˂ 0.0001, p˂0.0001). When compared to Citi 500 alone, the inclusion of *Lactobacillus* exhibited a more significant inhibitory effect on TNF-α (p ˂0.0001). Additionally, there is a substantial difference in TNFα-inhibition between Citi 500 and Citi 250 (p ˂0.001) (Fig. 3b).

### Effect of citicoline and its combination with *lactobacillus* on Nrf2 and HO-1

An essential upstream regulator of the expression of antioxidant enzymes, including HO-1, is Nrf2. The levels of Nrf2 and HO-1 in the livers of the NASH group significantly decreased as shown in Fig. 3c, d. As opposed to the NASH group, citicoline treatment either alone or in combination with *Lactobacillus* significantly increased Nrf2 and HO-1 expression in a dose-dependent manner. Additionally, when *Lactobacillus* was added compared to Citi 500 alone, HO-1 activation significantly increased (p 0.001) (Fig. 3c, d).

### Effect of citicoline and its combination with *Lactobacillus* on Caspase-3 and Bax

**Fig. 4:**
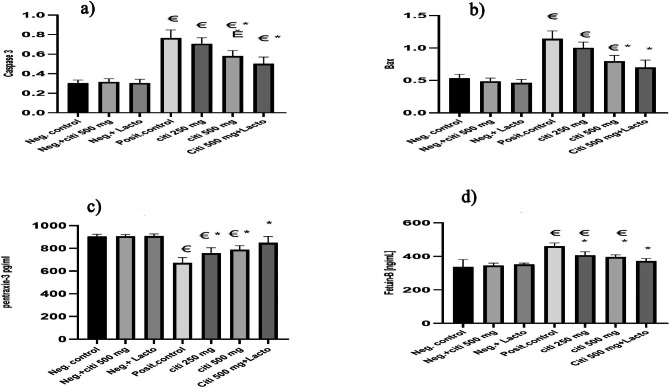
Effect of the tested monotherapies and therapeutic combinations on level of: (**a**) Caspase3, (**b**) Bax proteins, (**c**) pentraxin-3 (pg/mL), (**d**) Fetuin (ng/mL). Data are expressed as means ± SEM. Results are considered significant when P < 0.05. **Neg. control**: Control group; Neg.+ citi 500: Control + citicoline 500 mg group; Neg.+ Lacto: Control + Lactobacillus; Posit. control: HFD (Fatty liver) group, citi 250 mg: HFD + citicoline 250 mg group; citi 500 mg: HFD + citicoline 500 mg group; citi + Lacto: HFD + citicoline 500 mg + *Lactobacillus* group. *vs positive group, € vs. negative group, Ě vs. citicoline 250 mg, © vs. citicoline 500

Citi 500 significantly inhibits caspase 3 and Bax when used alone or in conjunction with *Lactobacillus* (p ˂0.001, p ˂0.0001, respectively). Additionally, the levels of caspase 3 change significantly between Citi 250 and Citi 500 (p ˂0.001) (Fig. 4a, b).

### Effect of citicoline and its combination with *Lactobacillus* on serum pentraxin-3 (pg/mL) and Fetuin (ng/mL)

Citicolline and its combination with *Lactobacillus* exhibit significant ameliorative effects on pentraxin (p ˂0.01, p ˂0.01, p ˂0.0001) and fetuin (p ˂0.01, p ˂0.01, p ˂0.001) serum levels, as shown in Fig. 4c, d, respectively. Citicoline has a dose-dependent impact.

### Effect of citicoline and *Lactobacillus* on lipid profile

**Fig. 5:**
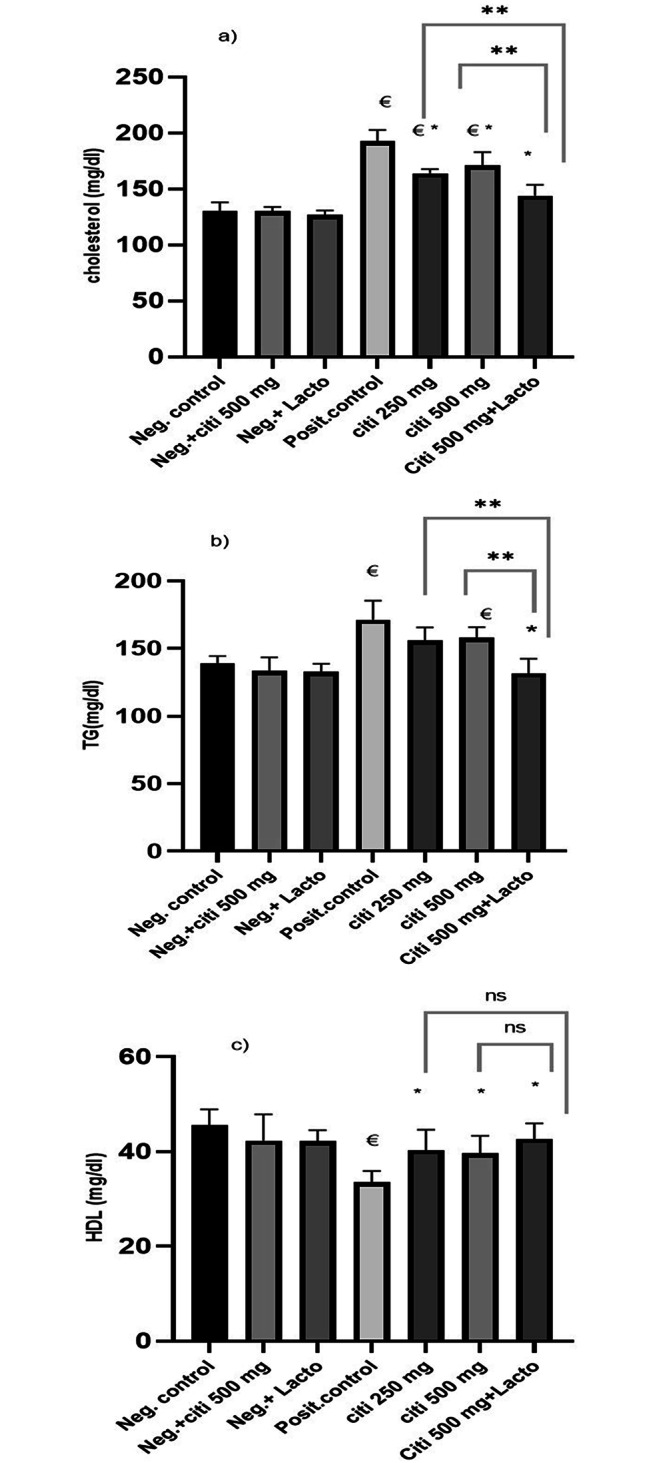
Effect of the tested monotherapies and therapeutic on serum level of lipid profile: (**a**) Cholesterol (mg/dl), (**b**) TG (mg/dl), (**c**) HDL (mg/dl). Data are expressed as means ± SEM. Results are considered significant when P < 0.05. Neg. control: Control group; Neg.+ citi 500: Control + citicoline 500 mg group; Neg.+ Lacto: Control + Lactobacillus; Posit. control: HFD (Fatty liver) group, citi 250 mg: HFD + citicoline 250 mg group; citi 500 mg: HFD + citicoline 500 mg group; citi + Lacto: HFD + citicoline 500 mg + *Lactobacillus* group. *vs positive group, € vs. negative group, Ě vs. citicoline 250 mg, © vs. citicoline 500

When the NASH group is compared to the negative control group, the level of cholesterol significantly increases. (P˂ 0.0001). All of the treatment groups’ cholesterol levels dramatically dropped, including Citi 250 (p ˂0.001), Citi 500 (p ˂0.001), and Citi plus Lacto (p ˂0.0001). Comparing Citi 500 to combined Citi 500 and *Lactobacillus* group shows a substantial improvement (p ˂0.001) (Fig. 5).

Only the combination of Citi 500 and Lacto had a significant inhibitory effect on TG when compared to the positive group (p ˂0.001). Adding Lacto to Citi 500 improved TG significantly when compared to Citi 500 alone (p ˂0.001). In terms of high-density lipoprotein (HDL), all treated groups have a significant increase (p ˂0.001, p ˂0.001, p ˂0.0001) of HDL-c compared to positive control. However, increasing citicoline dose or addition of *Lactobacillus* didn’t confer significant increase of HDL-c when compared to citi 250 group (Fig. 5).

### Effect of citicoline and its combination with *Lactobacillus* on hepatic histopathology

**Fig. 6:**
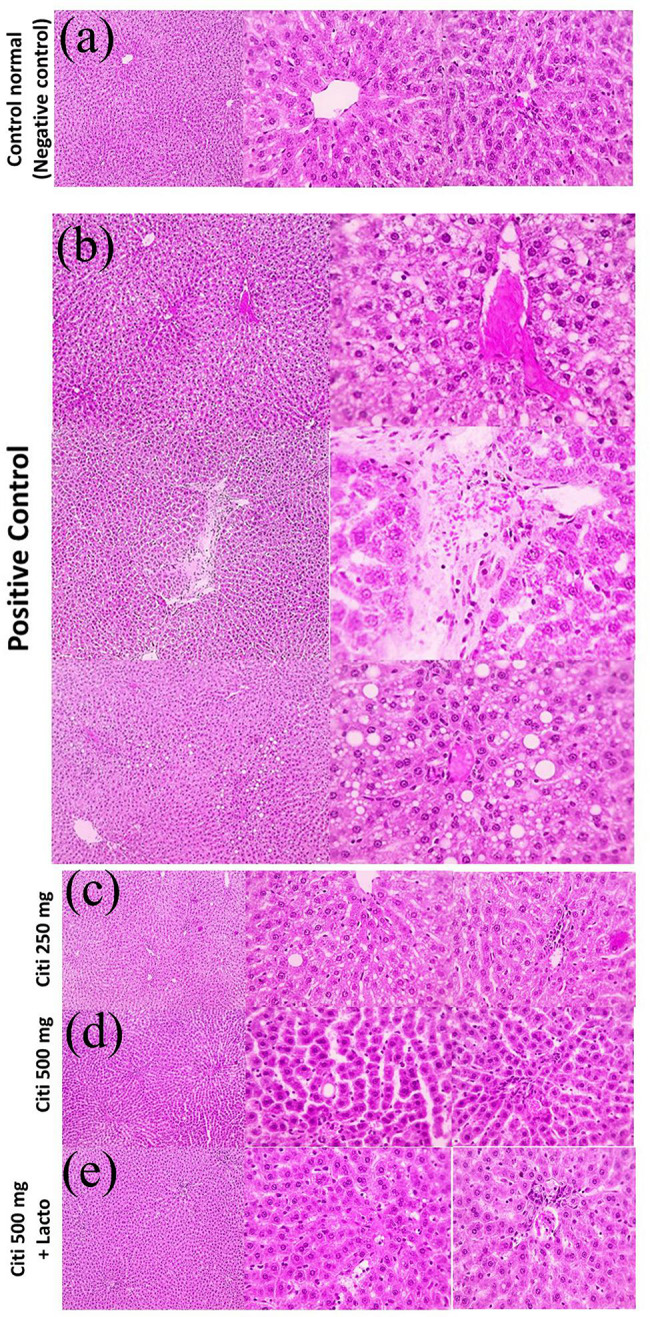
Microscopic pictures of H&E stained liver sections showing normal hepatocytes arranged in radiating plates around central vein (CV) with normal portal areas (PA) and sinusoids in control –ve group. Meanwhile, liver sections from NASH group showing congested blood vessels (red arrow), occluded sinusoids, diffuse hydropic degeneration with macro-vesicular steatosis (arrowheads). Portal fibrosis appears in some sections with foci of hemorrhage (red arrowhead) and few leukocytic cells infiltration (black arrows). Liver sections showing milder pathological changes in the treated groups including: few micro-vesicular steatosis (black arrowheads), mild micro- vesicular (blue arrowheads) intermingled with few macro-vesicular (black arrowheads) steatosis, in C250 group, few macro-vesicular steatosis (black arrowheads) in C500 group, very few micro-vesicular steatosis (blue arrowheads) in C500 + Lacto group. Low magnification X: 100 bar 100, high magnification X: 400 bar 50

**Fig. 7:**
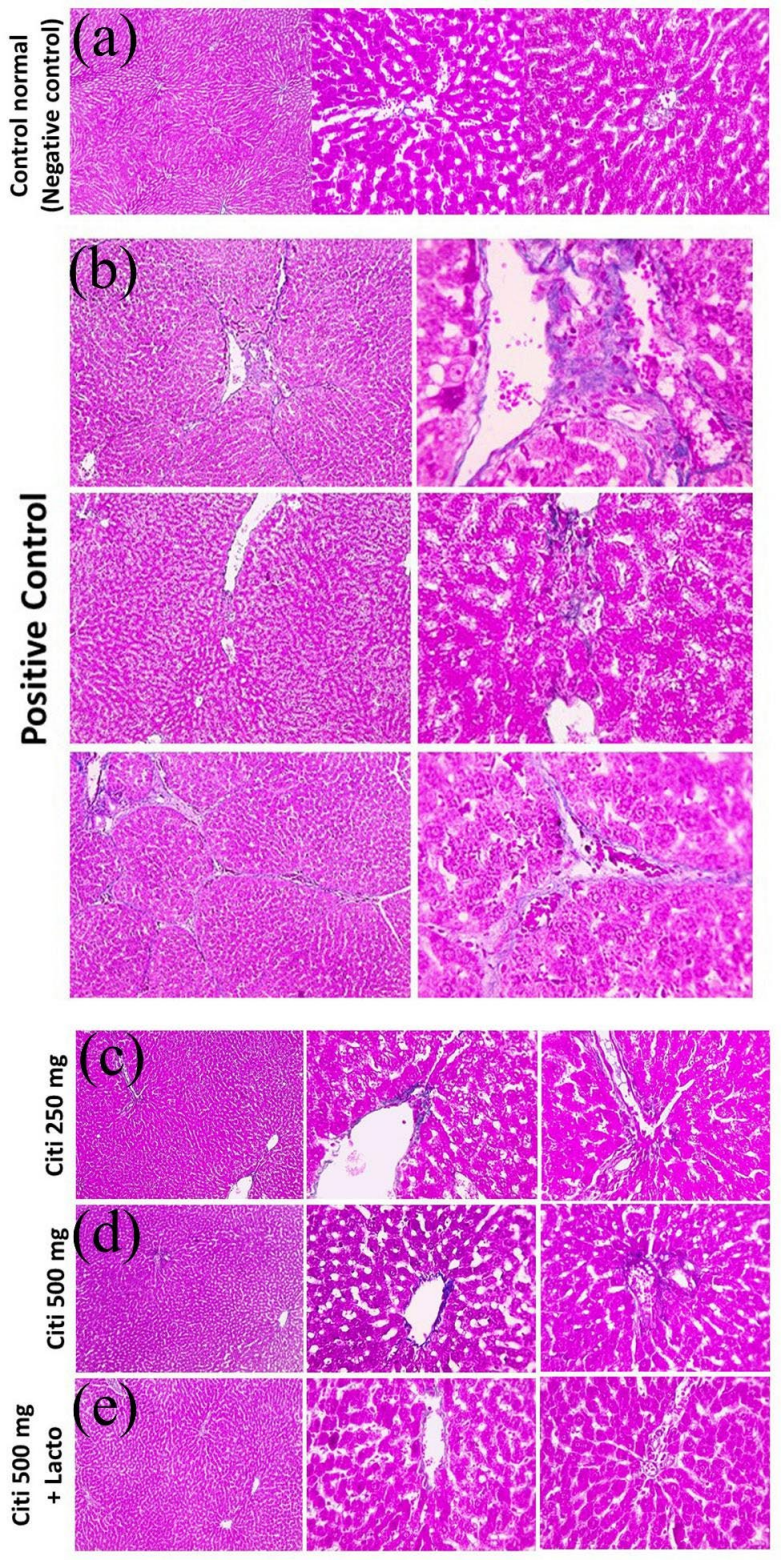
Microscopic pictures of Masson’s trichrome stained liver sections showing no excess collagen deposition around central vein (CV) or in portal areas (PA) in control –ve group. Meanwhile, liver sections from NASH group showing excess bluish stained collagen deposition around central vein (CV) or portal areas (PA) (black arrows). Liver sections from the treated groups showing decreased bluish stained collagen deposition around central vein (CV) or portal areas (PA) (black arrows) in C250 group, much less bluish stained collagen deposition around central vein (CV) or portal areas (PA) (black arrows) in C500 group, no excess bluish stained collagen deposition around central vein (CV) or in portal areas (PA) in C500 + Lacto group. Low magnification X: 100 bar 100, high magnification X: 400 bar 50

Microscopic pictures of H&E stained liver sections showed normal hepatocytes arranged in radiating plates around central vein (CV) with normal portal areas (PA) and sinusoids in negative control group. Meanwhile, liver sections from NASH group showed congested blood vessels, occluded sinusoids, diffuse hydropic degeneration with macro-vesicular steatosis. Portal fibrosis appears in some sections with foci of hemorrhage and few leukocytic cells infiltration. Milder pathological changes were observed in the treated groups including few micro-vesicular steatosis intermingled with few macro-vesicular steatosis in C250 group, few macro-vesicular steatosis in C500 group and very few micro-vesicular steatosis in C500 + Lacto group (Fig. 6). According to the observations from Mallory’s trichrome stained sections, negative control group showed no excess collagen deposition. However, liver sections from NASH group showed excess collagen deposition around central vein or portal areas. Liver sections from citicoline treated groups showed decreased collagen deposition in a dose dependent manner with lower collagen deposition on concomitant administration of *Lactobacillus* (Fig. 7).

### Effect of citicoline and its combination with *Lactobacillus* on hepatic expression of TLR-4 and NF-kB

**Fig. 8:**
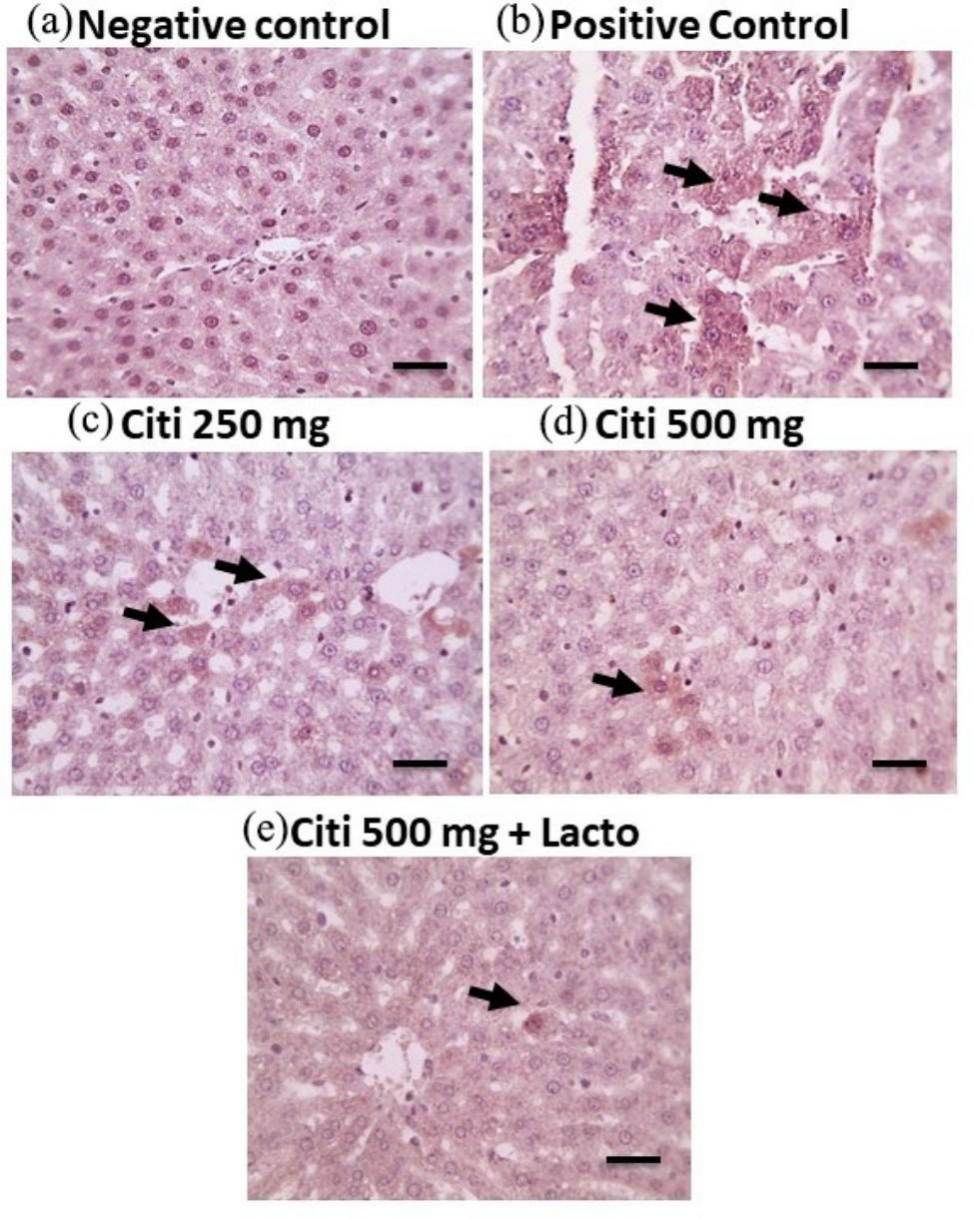
Microscopic pictures of immunostained liver sections against TLR showing negative staining in control –ve group. Meanwhile, liver sections from NASH group showing significant positive brown cytoplasmic staining in many hepatocytes (black arrows). Liver sections from the treated groups showing decreased positive brown cytoplasmic staining that appears in some hepatocytes (black arrows) in C250 group, much less positive cytoplasmic staining that appears in few hepatocytes (black arrows) in C500 group, very mild positive brown cytoplasmic staining that appears in very few hepatocytes (black arrows) in C500 + Lacto group. IHC counterstained with Mayer’s hematoxylin. Low magnification X: 100 bar 100, high magnification X: 400 bar 50

Regarding TLR-4 immunoreactivity, very few positively stained hepatocytes were found in normal rat livers. On the contrary, rats in NASH group showed a significant increase in the number of brownish stained hepatocytes as compared to the normal group. Treatment with citi 250, Citi 500 and further addition of *Lactobacillus* ameliorated TLR-4 expression as demonstrated by decreased positive brown cytoplasmic staining that appears in fewer hepatocytes respectively (Fig. 8).

**Fig. 9:**
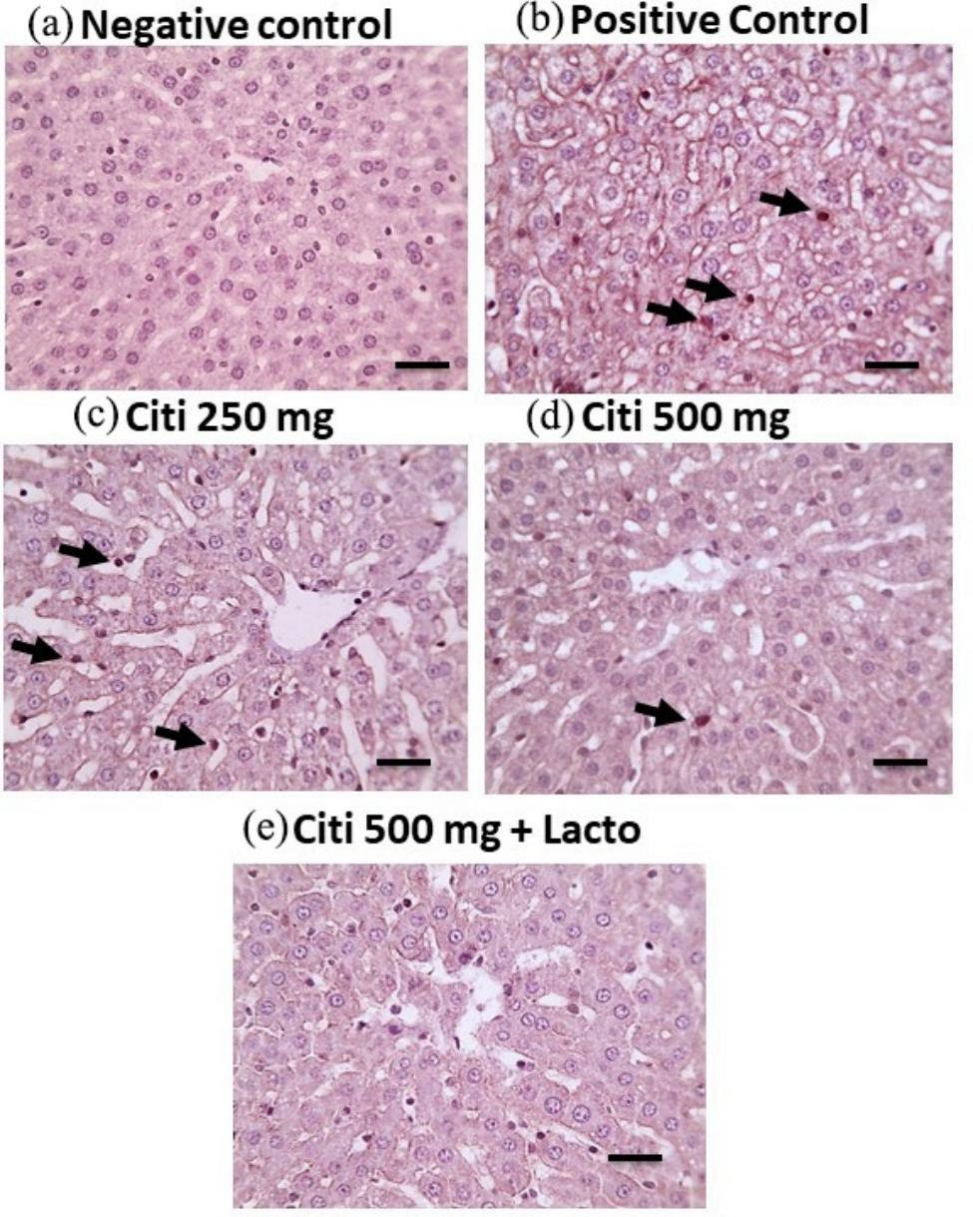
Microscopic pictures of immunostained liver sections against NF-kb showing negative staining in control –ve group. Meanwhile, liver sections from NASH group showing prominent positive brown nuclear staining of many von kupffer cells (black arrows). Liver sections from the treated groups showing decreased positive brown nuclear staining that appears in some von kupffer cells (black arrows) in C250 group, much less positive brown nuclear staining that appears in few von kupffer cells (black arrows) in C500 group, negative staining of von kupffer cells in C500 + Lacto group. IHC counterstained with Mayer’s hematoxylin. Low magnification X: 100 bar 100, high magnification X: 400 bar 50

On the other hand, immunostaining of NF-kB showed prominent positive brown nuclear staining of many von kupffer cells in liver sections from NASH group. As expected, a dose-dependent reduction in NF-kB was observed in the groups treated with citicoline, and to a greater extent on addition of *Lactobacillus*, compared to the NASH group (Fig. 9).

### Effect of citicoline and its combination with *Lactobacillus* on gut microbiota abundance

**Fig. 10:**
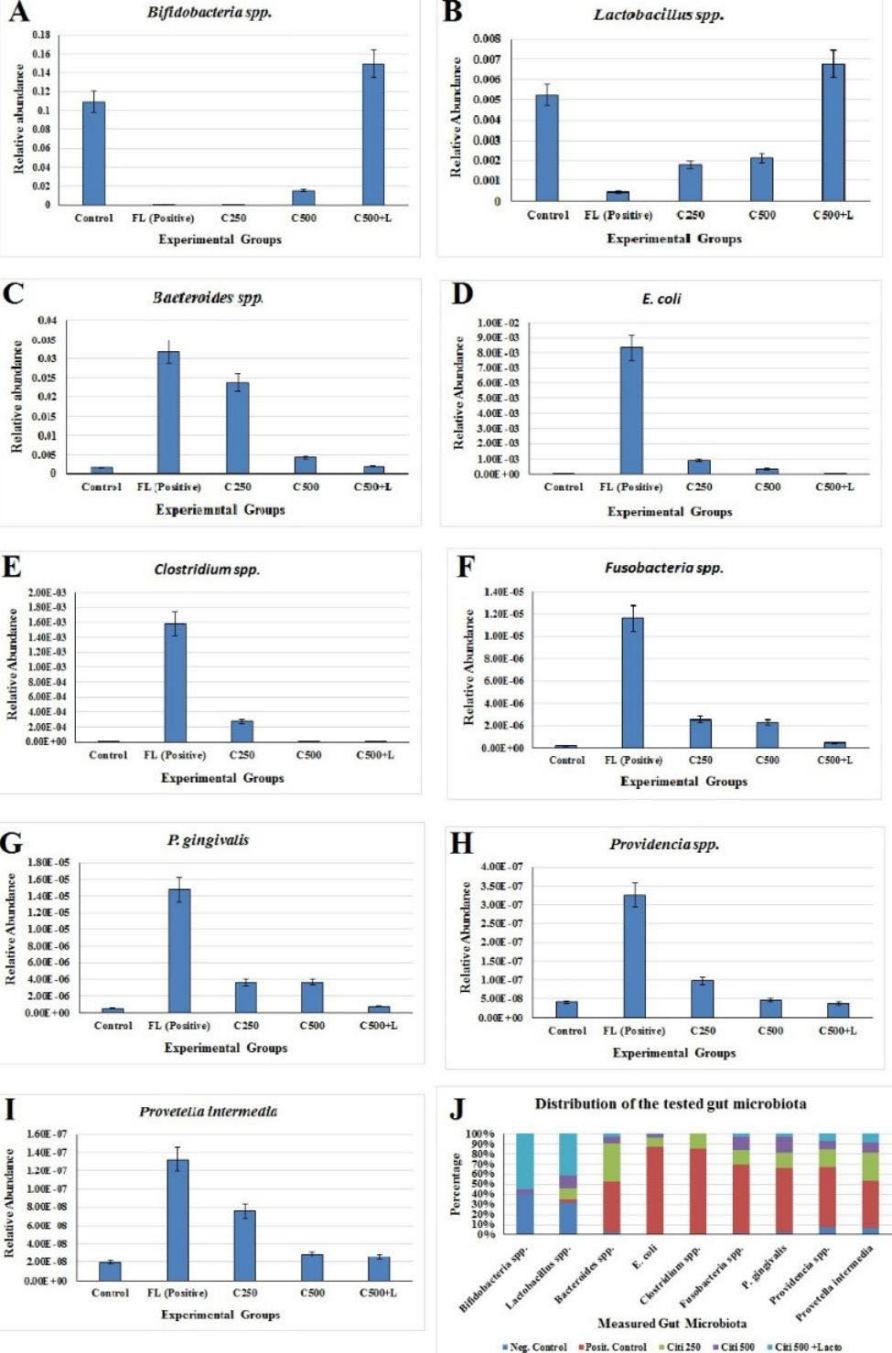
Relative abundance (RA) of different microbial species in different groups of tested samples in fatty liver animal model. RA is calculated for different groups of tested samples through targeted genes specific to different reference microbiota relative to the control housekeeping 16 S rRNA gene. (**A**) *Bifidobacteria* spp., (**B**) *Lactobacillus* spp., (**C**) *Bacteroides* spp., (**D**) *E. coli*, (**E**) *Clostridium* spp., (**F**) *Fusobacteria* spp., (**G**) *P. gingivalis*, (**H**) *Providencia* spp., (**I**) *Prevotella intermedia*, and (**J**) Distribution of the tested gut microbiota. Neg. control, Posit. control, citi 250 mg, citi 500 mg, and citi + Lacto represents different groups listed in the material and method section

In the current study, an alteration in gut microbiota was observed across the tested groups. A substantial difference in the relative abundance of microbiota, with NAFLD being linked to a considerable drop in *Bifidobacteria spp*. and *Lactobacillus spp.* compared to healthy controls. In comparison to a normal healthy control group, there was a massive rise in *Bacteroides spp., Fusobacterium spp., E. coli, Clostridium spp., Providencia spp., Prevotella interrmedia*, and *P. gingivalis*. In this study, administration of combined citicoline and *Lactobacillus* probiotics dramatically increased gut microbiota symbiosis when compared to the positive control group (Fig. 10).

## Discussion

NAFLD is the most common chronic liver disease worldwide. NAFLD is generally benign and non-progressive, whereas NASH can lead to cirrhosis and HCC. In terms of pathology, NASH includes not only liver steatosis but also hepatic inflammation and/or fibrosis (Clapper et al. 2013). The first-line treatment for NAFLD is a weight-loss lifestyle strategy (Promrat et al. 2010). Although numerous pharmaceutical alternatives for NASH therapy are employed (insulin sensitizers, lipid-lowering medications, and antioxidants), they either have modest effects or have long-term negative impact that limit their usage (M et al. 2009). Till now, there is no effective medicine for NASH; therefore, it is vital to search for an effective therapy. During the past few years, several researchers suggested that dysbiosis of intestinal microbiota could contribute to NAFLD occurrence and progression (Aragonès et al. 2019; Mokhtari et al. 2017). Hence, management of gut microbiota may be a promising strategy in NAFLD therapy. In this work, the effects of citicoline and its combination with lactobacillus in HFD and STZ-induced NASH, as well as the underlying mechanisms were investigated in a murine animal model.

Several studies reported that administration of a mixture of probiotics including *Lactobacillus* spp. has been proven to alleviate NAFLD symptoms in animal models (Esposito et al. 2009; Li et al. 2003; Ma et al. 2008; Nardone et al. 2010; Velayudham et al. 2009). In this study, citicoline administration alone or in combination with lactobacillus significantly reduced body and liver mass as well as hepatic index compared to untreated NASH group. Reduced net calorie intake during citicoline medication may be responsible for weight reduction, most likely due to appetite suppression (Killgore et al. 2010). Additionally, the gut flora plays a significant role in controlling body weight by either influencing energy metabolism or preventing the absorption of lipids (Ley et al. 2006). As a result, the Lactobacillus/Citicoline combined regimen had an advantage in reducing liver mass and liver index, which may be connected to a decrease in the deposition of fat in the liver and a corresponding decline in liver index.

The current findings demonstrated dyslipidemia in NASH group, as evidenced by elevated cholesterol and TG levels in the serum, as well as reduced HDL-C serum levels. These negative consequences have been demonstrated in previous studies (Afrin et al. 2017; Gerges et al. 2020). Notably, citicoline treatment improved lipid profile. The addition of lactobacillus in concomitant with citicoline induced an additional decrease in cholesterol and TG levels as well as increase in HDL-C. Similarly, a previous study reported that probiotics significantly improved serum TG and LDL levels in animal model (Al-Muzafar and Amin 2017). Furthermore, it was reported that liver steatosis can be ameliorated with IV-choline-supplement (Buchman et al. 1995).

Furthermore, the possible molecular pathways involved in the therapeutic effects of citicoline and lactobacillus were investigated. Oxidative stress is a major cause of hepatic injury in NASH according to animal models and individuals with the disease. Cytochrome P450, peroxisomal β-oxidation, mitochondrial electron leak, and recruited inflammatory cells have all been identified as contributors of oxidative stress in fatty liver (Machado et al. 2008; Mitsuyoshi et al. 2006). In mitochondria, ROS are produced during the metabolism of free fatty acids, reactive lipid peroxidation products such as MDA can further aggravate oxidative stress, leading to the development of new sources of oxidants. The current findings confirmed the accumulation of MDA (oxidative stress marker), in rats with NASH, along with low SOD and TAC levels. The increase in oxidative stress provoked liver injury, as shown in this study by elevation of serum ALT, AST and ALP levels. These results were also supported by the histopathological findings in which diffuse hydropic degeneration of hepatocytes was observed along with macro-vesicular steatosis, central vein congestion and leukocytic cells infiltration in NASH group. However, the use of citicoline alone or in combination with lactobacillus improved the antioxidant system and restored SOD and TAC levels, resulting in reduced hepatocyte lipid peroxidation with modest improvement of liver function markers and histopathological abnormalities. These findings are in accordance with a recent study reporting that administration of probiotics improved oxidative stress markers in NAFLD animal model (Azarang et al. 2020). It was also reported that probiotic therapy significantly reduce liver specific markers such as ALT, AST and total cholesterol (Ma et al. 2013; Sharpton et al. 2019). Many investigations have shown that citicoline has a strong inhibitory effect on the production of reactive oxygen species (ROS) (Al-Kuraishy and Al-Gareeb 2020; Aminzadeh and Salarinejad 2019; Qian et al. 2014).

Nrf2 is a redox-sensitive transcription factor that regulates detoxification genes and cellular responses to oxidative stress. Nrf2 is required for protection against oxidative liver damage in NASH mouse models (Chowdhry et al. 2010; Okada et al. 2013). Nrf2 is released and translocated into the nucleus in response to oxidative stress, where it stimulates the expression of many antioxidant genes such as HO-1 (Ma 2013). Overexpression of HO-1 can protect cells against oxidative stress-induced cell death (Min et al. 2011). Nrf2 activators are being developed for the treatment of NASH (Musso et al. 2016). In line with this, our findings revealed that NASH rats showed decreased expression and activity of both Nrf2 and its target genes. On the other hand, therapy with citicoline either alone or in combination with lactobacillus increased Nrf2 and its target gene expression. These findings lead us to believe that citicoline protective effects may be mediated by activation of the Nrf2 pathway, which boosts the liver’s antioxidant capacity.

The GLA mediates NASH progression; Altered gut microbiota is associated with increased production of microbial metabolites such as LPS which is transported to the liver via portal vein (Jiang et al. 2020). It was reported that LPS/toll-like receptor 4 (TLR4)/NF-kB signaling is critical for the activation of inflammatory pathways associated with NASH (Machado and Cortez-Pinto 2012). TLR4 binding with LPS cause NF-kB activation and TNF-α production (Neuman et al. 2012). NF-kB signaling is also activated in response to oxidative stress and regulates inflammation and immunological responses (El-Agamy et al. 2019). NF-kB promotes the release of inflammatory cytokines such IL-6 and TNF-α causing liver tissue damage and increasing NAFLD aggravation (Henkel et al. 2018; Zhang et al. 2016). In line with the aforementioned studies, the results of our investigation indicated an increase in TLR4 expression, NF-kB activation and subsequent release of inflammatory cytokines (TNF-α, IL-6) in the NASH group with marked inter-parenchymal lymphocytes in the histological examination. Citicoline, on the other hand, ameliorated the activated inflammatory pathway, LPS/TLR4/NF-κB/TNF-α and IL-6, demonstrating that citicoline had a significant anti-inflammatory impact. Concomitant administration of lactobacillus caused further improvement of inflammatory markers with only mild lobular and portal inflammation. These findings revealed that suppression of TLR4/NF-kB was involved in citicoline and lactobacillus hepato-protection in NASH. Our findings supported by prior research that indicated citicoline suppressed the release of inflammatory cytokines (Bogdanov et al. 2018; Ek et al. 2014; Masoud et al. 2021). In addition, several studies reported that probiotics including lactobacillus inhibited inflammatory signaling such as NF-κB, reduced serum LPS and liver TLR4 expression and delay NAFLD progression (Li et al. 2003; Nagashimada and Honda 2021; Xue et al. 2017).

Patients with NASH have high plasma levels of TNF-α and PTX-3 (Pearce et al. 2013). When inflammatory cytokines are stimulated, PTX3 is rapidly produced in diverse cells (Bottazzi et al. 2009). A greater fibrosis grade was linked to high plasma levels of PTX3 (Boga et al. 2015). PTX-3 expression contributes to increased synthesis of extracellular protein matrix, suggesting a possible role in liver fibrosis (Feder et al. 2020). In the current study, increased levels of PTX3 and TNF-α were observed in liver tissues, which coincided with histological analysis of stained liver tissues showing excess collagen deposition around central vein and portal areas, validated these prior findings. Citicoline, interestingly, reduced the levels of PTX3 and TNF-α and improved the fibrosis score. Combined regimen with lactobacillus caused further improvement. This matches a prior study reporting that citicoline therapy lowers fibrosis and fibroblast cell density in a rat model of laminotomy and discectomy (Savran et al. 2012). Furthermore, it was reported that probiotic consumption significantly reduced the levels of serum PTX3 (Pourrajab et al. 2020). In addition, TNF-α and IL-6 levels reduced significantly after using probiotics in NAFLD patients (Kobyliak et al. 2018).

High serum level of fetuin was linked to NAFLD (Zhu et al. 2017). Fetuin B is a hepatokine that is upregulated by hepatic steatosis in obese persons and increased in type 2 diabetes (Meex et al. 2015). Furthermore, fetuin B had a positive relationship with serum ALT, TG and LDL-C, and a negative relationship with serum HDL-C (Zhu et al. 2017). This is consistent with our findings, which indicated high serum fetuin B levels in NASH rats. Treatment with citicoline alone or in combination with lactobacillus reduced this rise, indicating their therapeutic impact in NASH.

Apoptosis is a programed cell death that is induced by several factors including oxidative stress. Bcl2, Bax, and caspase-3 are the most common and reliable apoptosis markers (Elmore 2007). Hepatocyte apoptosis plays a significant role in liver injury (Wieckowska et al. 2006). Previous research has connected NASH- liver apoptosis to increased expression of caspases-3 and Bax (Wang et al. 2008). Furthermore, Nrf2 and NF-kB signaling pathways, which contribute to hepatocyte death, have been related to changes in pro-apoptotic proteins in NASH mice (Sharma et al. 2018). In the current study, apoptosis is elevated in the liver of NASH rats, which is indicated by increased levels of Bax and caspase-3. Citicoline, interestingly, reduced HFD-induced changes in these apoptotic markers. These findings coincides with a previous study stated that citicoline alone and in combination with hypothermia is effective in ameliorating cerebral damage after transient focal ischemia by suppressing apoptotic processes as indicated by increased Bax and Caspase-3 (Sahin et al. 2010). On the other hand, addition of lactobacillus further depressed apoptotic markers. Consistent with this, pretreatment with *Lactobacillus* diminished the levels of Bax and caspase 3 in hepatic cell lines (Kanmani and Kim 2022). These findings support the hypothesis that citicoline alters the Nrf2/NF-kB pathways, suppress hepatocyte apoptosis and improves hepatic damage.

In the current study high level of *Lactobacillus* and *Bifidobacterium* were observed in normal group compared to positive (fatty liver) group. It was reported that HFD increased intestinal endotoxin, and reduced enteric concentration of Bifidobacteria (Xu et al. 2011). After treatment with *Lactobacillus* and citicoline the levels of both *Bifidobacterium* and *Lactobacillus* increased to be above the normal group. Similar to this, a prior study showed that giving rats fed a high-cholesterol diet *Lactobacillus* decreases both cholesterol and TG while increasing the amount of faecal *Lactobacilli* and *Bifidobacteria*. (Wang et al. 2009).

This study reported overgrowth of *Bacteroides, E. coli, Fusobacterium, Providencia, Clostridium* and *Prevotella* during NAFLD that restored to normal level after treatment with *Lactobacillus* and citicoline. Consistent with our results, it was reported that Proteobacteria including *Escherichia coli* were significantly elevated in NASH (Zhu et al. 2013). In addition, Bacteroides overgrowth was reported recently in NASH rat model (Carter et al. 2021), and in a study involving human subjects (Boursier et al. 2016). Stool microbiome of children with NAFLD showed high abundance of *Prevotella* (Michail et al. 2015). Furthermore, Fusobacteria phyla were more abundant in NAFLD patients than healthy individuals (Shen et al. 2017). In addition, a retrospective study reported that NAFLD is a risk factor of *Clostridium difficile* GIT infection (Nseir et al. 2020).

Although *P. gingivalis* is a periodontal pathogen, it was reported that periodontal pathogens can cause changes in gut microbiota. *P. gingivalis* is acid resistant, so it can pass through the stomach and reach the intestinal tract (Nakajima et al. 2015). Variation in the gut microbiota was seen as periodontal infections moved to the intestine, for example, Firmicutes decreased and Bacteroides increased (Liu et al. 2021). All of this is consistent with the elevation of *P. gingivalis* observed in fatty liver group in the current study. *P*. *gingivalis* infection was found to be an important risk factor for progression of liver fibrosis in NAFLD (Nakahara et al. 2018). Bacteroides and *E. coli* reach high abundance during liver fibrosis (Ji et al. 2020).

Overall, our data show that citicoline either alone and in combination with a mixture of *L. fermentum* and *L. delbruceki* have strong anti-inflammatory and antioxidant properties that are effective against NASH. These properties may be related to Nrf2/HO-1 pathway activation and TLR4/NF-kB mediated inflammatory pathway inhibition, which may attenuate NASH associated altered lipid profile, liver damage, and apoptosis. They are therefore potential new hepatoprotective options for NASH.

## Electronic supplementary material

Below is the link to the electronic supplementary material.

Supplementary Material 1

## Data Availability

Enquiries about data availability should be directed to the authors.

## Abbreviations

NASH: Non-alcoholic steatohepatitis
NAFLD: Non-alcoholic fatty liver disease
HFD: High-fat diet
STZ: Streptozotocin
MDA: Malondialdehyde
GSH: Glutathione
TAC: Total antioxidant capacity
TNF-α: Tumor necrosis factor alpha
IL-6: Interleukin 6
Nrf2: Nuclear factor erythroid 2
HO-1: Heme oxygenase-1
TLR4: Toll-like receptor 4
NF-kB: Nuclear factor kappa B
AST: Aspartate aminotransferase
ALT: Alanine aminotransferase
ALP: Alkaline phosphatase
SOD: Superoxide dismutase
HCC: Hepatocellular carcinoma
PTX3: Pentraxin 3
TC: Total cholesterol
TGs: Triglycerides
HDL-C: High-density lipoprotein cholesterol.
