# Supplementary figures and images for: Valuable effects of lactobacillus and citicoline on steatohepatitis: role of Nrf2/HO-1 and gut microbiota
Source: AMB Express. 2023 Jun 8;13:57. doi: 10.1186/s13568-023-01561-8 (PMC10250290; doi:10.1186/s13568-023-01561-8)

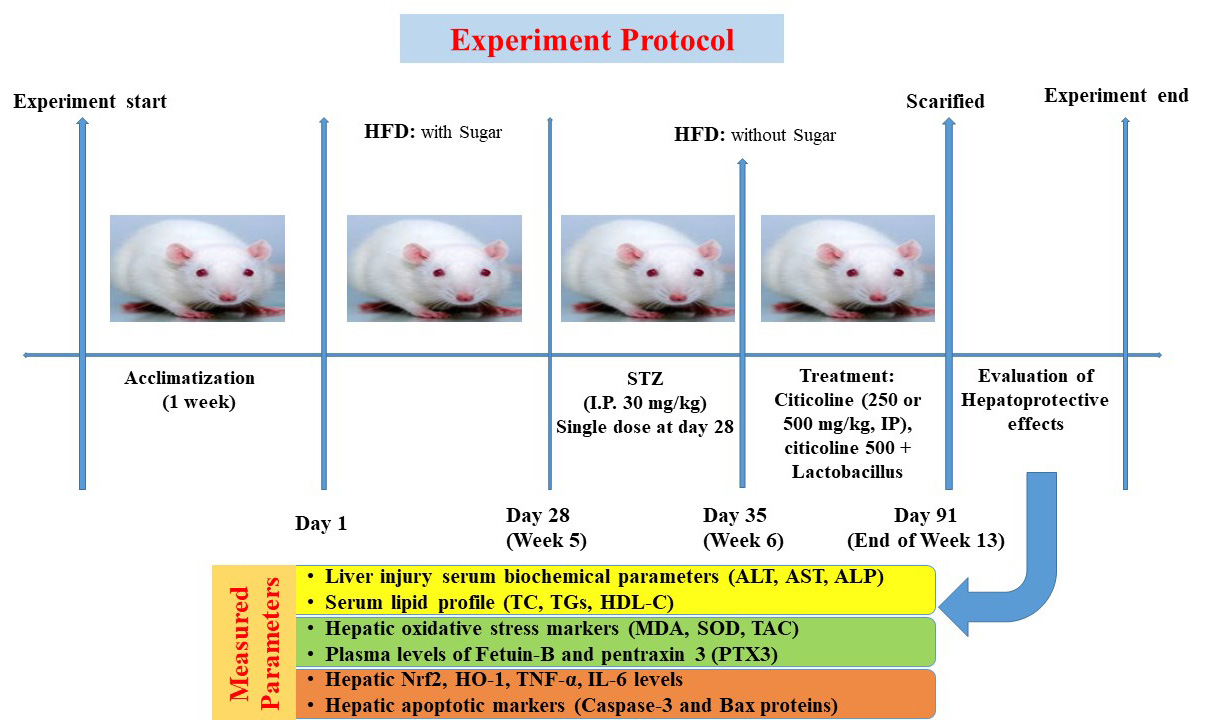

Supplement: Supplementary file 1 — Supplementary Material 1 [file 13568_2023_1561_MOESM1_ESM.jpg]
